# Liver Stiffness Measurement among Patients with Chronic Hepatitis B and C: Results from a 5-Year Prospective Study

**DOI:** 10.1371/journal.pone.0111912

**Published:** 2014-11-04

**Authors:** Karen M. Christiansen, Belinda K. Mössner, Janne F. Hansen, Erik F. Jarnbjer, Court Pedersen, Peer B. Christensen

**Affiliations:** Department of Infectious Diseases, Odense University Hospital, Odense, Denmark; Kanazawa Medical University, Japan

## Abstract

Liver stiffness measurement (LSM) is widely used to evaluate liver fibrosis, but longitudinal studies are rare. The current study was aimed to monitor LSM during follow-up, and to evaluate the association of LSM data with mortality and liver-related outcomes. We included all patients with chronic viral hepatitis and valid LSM using Fibroscan. Information about liver biopsy, antiviral treatment, and clinical outcome was obtained from medical records and national registers. The study included 845 patients: 597 (71%) with hepatitis C virus (HCV), 235 (28%) with hepatitis B virus (HBV) and 13 (2%) with dual infection. The initial LSM distribution (<7/7–9.9/10–16.9/≥17 kPa) was 58%/16%/14%/12%. Among patients with initial LSM values of 7–9.9 kPa, 60% of HCV patients and 83% of HBV patients showed LSM values of <7 kPa at the latest follow-up. Progression rates (defined as >20% and >2 kPa increase, with one measure >7 kPa) were 3.4/100 person years (PY) for HCV and 1.5/100 PY for HBV infected patients. Patients with LSM values of ≥17 kPa had the same liver-related complication incidence as patients with biopsy-proven cirrhosis (11.1 versus 12.1/100 PY). Thirteen liver-related deaths occurred among HCV patients (0.6/100 PY), but none among HBV patients. Among patients who died of liver-related causes, all but one had baseline LSM values of ≥17 kPa. Overall, patients with LSM values <17 kPa were not associated with adverse outcomes. In contrast, LSM values ≥17 kPa were associated with significant risk of liver-related problems. The results of the current study suggest that clinical decisions should not be taken based on a single LSM measurement.

## Introduction

In patients with viral hepatitis, morbidity and mortality are closely related to fibrosis development, which can lead to cirrhosis and end-stage liver disease. Liver biopsy is considered the gold standard method for fibrosis diagnosis; however, this procedure carries rare but serious complications and substantial problems with sampling variability and observer agreement [1]. Thus, alternative non-invasive methods have been eagerly pursued for decades [2], [3].

Liver stiffness measurement (LSM) using the Fibroscan device from Echosens was originally introduced in clinical practice in 2003 [4]. This technique performs well in classifying patients at the extremes of no significant fibrosis (Metavir F0–F1) and cirrhosis (F4), but is less accurate for detecting significant fibrosis (F2–F3) [3]. Measurement can also be difficult in patients with narrow intercostal space and/or obesity, with invalid results reported in up to 20% of patients. Furthermore, several factors—including liver inflammation, right-sided heart failure, and measurement in the post-prandial state—may increase the LSM, thus overestimating the fibrosis present [5]–[8].

To date, there is no universal consensus regarding a normal LSM value, or how to define cut-offs for significant fibrosis and cirrhosis. LSM may vary with the etiology of liver fibrosis, as patients with hepatitis B virus (HBV) reportedly have lower LSM values than patients with hepatitis C virus (HCV) at the same stage of fibrosis [9]. Among blood donors with no signs of liver disease and in health care center-based screenings, median/95th percentile values have been reported to be 4.4/6.7 kiloPascal(kPa) and 5.3/8.0 kPa [10]–[12]. Two large meta-analyses determined cut-off values of 7.2/7.7 kPa for F2 and of 13.0/14.5 kPa for cirrhosis among patients with liver disease [13], [14]. However, a large French multicenter study identified 17 kPa as a reliable cut-off for cirrhosis. Current Danish national guidelines for patients with viral hepatitis recommend 17 kPa as the cut-off for cirrhosis and 7 kPa as the upper limit of normal [15], [16]. Liver-related morbidity and mortality is primarily seen in patients with cirrhosis, and LSM is significantly correlated with hepatic vein portal gradient (HVPG) [17], [18]. To better discriminate between patients with and without substantial fibrosis (F2), algorithms have been developed to combine LSM with serological markers, but no individual test or algorithm has yet attained universal acceptance [3], [19]. However, LSM is currently used in clinical practice in Europe and was approved for use in the US in 2013.

The present study aimed to evaluate LSM over time in a well-defined population of patients with chronic viral hepatitis B or C. The hypothesis was that high or increasing LSM values would be predictive of liver-related morbidity and mortality.

## Patients and Methods

This study included all patients whose first LSM was performed between May 1, 2007 (the date that our department began to implement LSM) and April 30, 2012. LSM examinations were performed using the Fibroscan 402 and M probe (Echosens, Paris, France) according to the manufacturer's guidelines. All examiners were certified by Echosens. In 2010, we introduced the use of the XL probe for obese patients, which was employed in 8% of all examinations. All measurements were automatically stored on the Fibroscan hard disk, with back-up on a central server. For the present study, personal ID (civil registration number) and LSM measurements were obtained from a file from the Fibroscan device. Vital status was obtained from the civil register at the end of observation (July 31, 2012), and causes of death were obtained from the register of death certificates. For 2012, information from the death certificate register was not yet available, and therefore causes of death were obtained from individual death certificates and hospital clinical records. Information about morbidity and antiviral treatment was obtained from hospital records.

This study was approved by the Local Committee on Biomedical Research Ethics (VF 20050089) and the Danish Data Protection Agency (2006-41-7196 and 2012-41-0079). Participants gave written informed consent.

Routine LSM was performed with no food restrictions. Patients with LSM above normal were offered a fasting LSM within 1–3 months. A valid LSM examination included 10 valid measurements, a success rate of 60%, and an interquartile range of measurements (IQR) below 30% of the median value. We considered an LSM of <7 kPa to be normal and an LSM of >17 kPa to indicate cirrhosis. Lacking previous guidelines to define a significant change in LSM over time, we applied the following definition: a 20% change in LSM value was regarded as significant if the observed change was ≥2 kPa and if at least one of the measurements was >7 kPa. The requirement of one absolute value above 7 kPa was included to avoid classifying trivial changes within the normal range as significant changes. This also compensated for the intra-individual stochastic variation observed by repeated measurements over short periods of time and for the precision of measurement (0.5 kPa). LSM was offered at least once yearly, and more frequently if deemed necessary. Patients with repeated LSM values in the range of 7–9 kPa were usually closely monitored, while patients with values of above 9 kPa were recommended to undergo biopsy or start treatment based on an overall evaluation. After 2010, patients with a confirmed LSM of >17 kPa were started on cirrhosis surveillance and treatment without biopsy.

Prior to 2007, cirrhosis was diagnosed based on liver biopsy, development of clinical decompensation (ascites, presence of esophageal varices with or without bleeding, encephalopathy, spontaneous bacterial peritonitis, or hepatorenal syndrome), or detection of hepatocellular carcinoma (HCC). Following the introduction of LSM, patients with LSM of >17 kPa were also classified as having cirrhosis. For incidence calculation, we excluded a lead-in phase of 6 months after the first LSM, in order to exclude the prevalent decompensation detected during the initial workup of an elevated first LSM.

The LSM natural history follow-up cohort included patients infected with HBV or HCV in whom two or more LSM were performed at least 3 months apart. We excluded patients with dual infection with HBV, HCV, and/or human immunodeficiency virus (HIV), and patients who were treated between January 1, 2006 and start of the inclusion period. Patients whose treatment started during follow-up (as part of the LSM natural history cohort) were censored at the date of last LSM before treatment initiation.

Statistical analysis was performed using Stata version 12. Nonparametric statistical tests (Fisher's exact test, chi-square test, and Wilcoxon rank-sum test, as appropriate) were employed, and results were reported with 95% confidence intervals (95% CI). A two-sided p value of <0.05 was considered significant.

## Results

**Figure 1:**
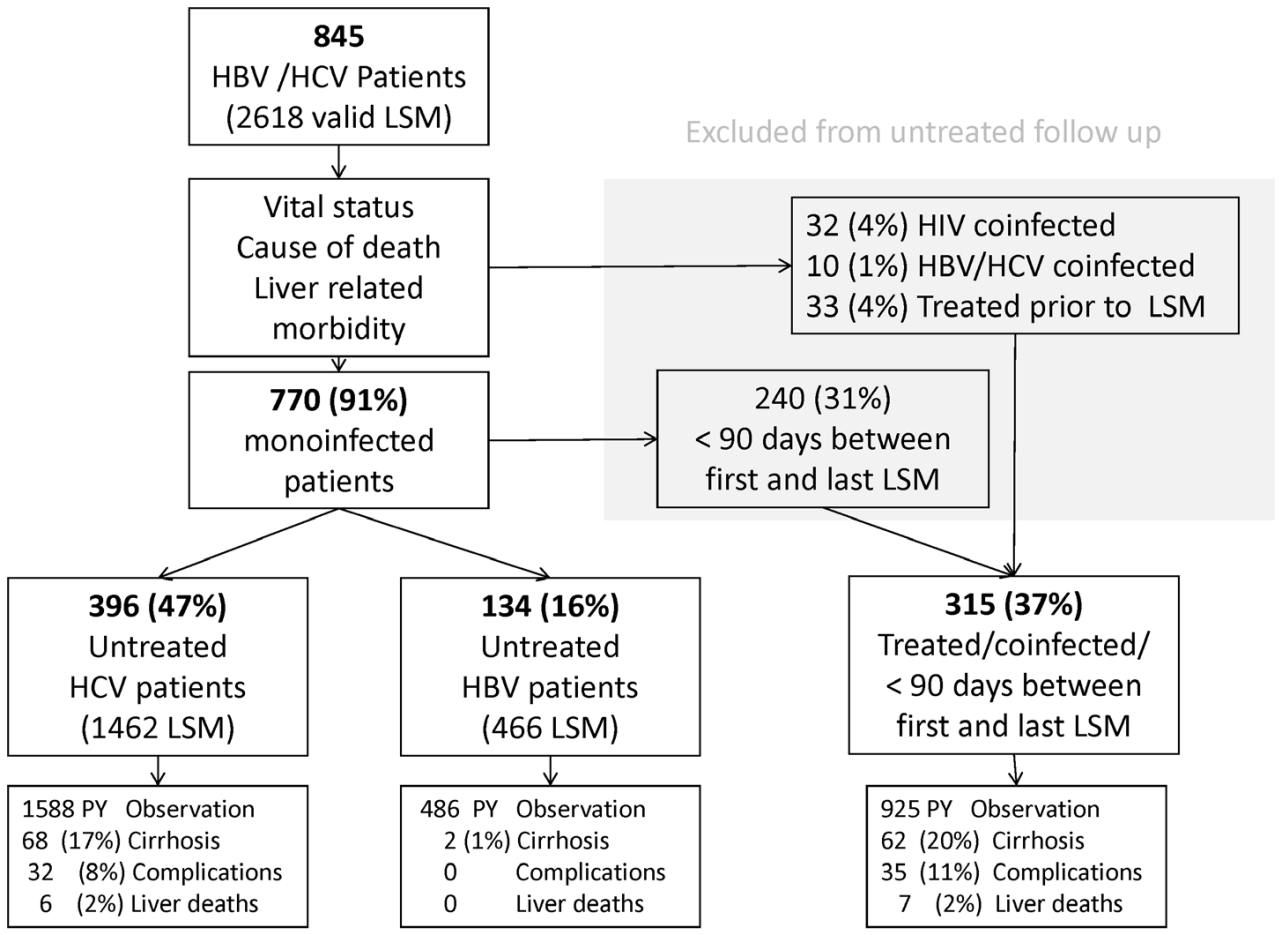
Flow chart of patients showing follow-up liver stiffness measurements.

The study included a total of 845 patients with chronic viral hepatitis and a valid LSM examination. Of these patients, 71% (597) had hepatitis C, 28% (235) hepatitis B, and 2% (13) had both infections (Figure 1). Hepatitis B and C patients significantly differed in many aspects, with hepatitis C patients more commonly showing fibrosis-associated factors, such as older age, male gender, alcohol consumption, etc. Median LSM was 1.8 kPa higher in hepatitis C patients than hepatitis B patients (p<0.001; Table 1). Several liver test results were also significantly higher among hepatitis C patients (Table 2).

**Table 1:** Demographics of study populations.

	HCV		HBV		HCV/HBV, all
	Untreated follow-up	All	Untreated follow-up	All	p value
Male gender, n (%)	246 (62)	381 (64)	61 (46)	111 (47)	<0.001
Age at first scan (years), median (IQR)	45 (36–52)	45 (36–52)	34 (29–43)	36 (29–47)	<0.001
Injecting drug use (ever), n(%)	248 (63)	374 (63)	1 (1)	3 (1)	<0.001
Self reporet alcohol overuse (ever), n(%)	187 (47)	248 (42)	12 (9)	20 (9)	<0.001
Baseline liver stiffness, median(IQR)	6.9 (5.6–10.5)	6.9 (5.4–11.8)	5.2 (4.2–7)	5.2 (4.3–6.8)	<0.001
Baseline liver stiffness ≥17 kPa, n (%)	46 (12)	94 (16)	1 (1)	7 (3)	<0.001
Liver biopsy Metavir score*					
Fibrosis, median (IQR)	1.5 (1–3)	1.5 (1–3)	1 (0.5–1)	1 (0–1)	0.041
Activity, median (IQR)	0.5 (0–2)	1 (0–2)	0 (0–1.5)	0 (0–2)	0.224
Liver Biopsy with cirrhosis, n (%)	29 (7)	47 (8)	0 (0)	2 (1%)	<0.001
Observed clical decompentsations, n (%)	32 (8)	61 (10)	0 (0)	4 (2)	<0.001
Observed total deaths, n (%)	27 (7)	50 (8)	0 (0)	5 (2)	<0.001
Liver related deaths, n (%)**	5 (1)	12 (2)	0 (0)	0 (0)	0.030
Sum of follow up in person years	1133	2198	0 (0)	744	
Total, n (%)	396 (100)	597 (100)	134	234 (100)	

These data exclude 13 patients co-infected with HBV and HCV, and include 29 patients co-infected with HIV.

* Liver biopsy data for 68 patients with biopsy within one year of liver stiffness measurement.

**Excluding 1 hepatitis C patient with an unconfirmed liver-related cause of death.

**Table 2:** Laboratory tests at first liver stiffness measurement.

		HCV		HBV		HCV/HBV
		Untreated follow-up	All	Untreated follow-up	All	All
	Normal range	Median (IQR)	Median (IQR)	Median (IQR)	Median (IQR)	p value
Alanine aminotransferase	10–50 U/L	54 (34–91)	54 (34–95)	32 (23–47)	33 (23–49)	<0.001
Asparatate aminotransferase	15–45 U/L	46 (33–75)	46 (33–76)	30 (25–36)	30 (25–36)	<0.001
Alkaline phosphatase	80–285 U/L	81(63–103)	81 (64–105)	71 (57–84)	74 (60–89)	<0.001
Gamma-glutamyltranferase	15–115 U/L	60 (28–119)	58 (28–117)	21 (14–32)	21 (15–38)	<0.001
Bilirubin	5–25 µmol/L	8 (6–11)	8 (6–12)	8 (6–12)	8 (6–12)	0.983
International normalized ratio	0.9–1.2	1.0 (0.9–1.1)	1.0 (0.9–1.1)	1.0 (1.0–1.1)	1.0 (1.0–1.1)	0.003
Albumin	36–48 g/L	44 (42–46)	44(41–46)	45 (43–47)	44 (42–46)	0.319
Platelets	145–350 109/L	224 (175–278)	222 (170–272)	228 (194–282)	226 (193–226)	0.203
Total, n (%)*		396 (100)	597 (100)	134 (100)	234 (100)	

Excluding 13 patients co-infected with HBV and HCV.

All tests were performed within three months of the first liver stiffness measurement, except for aspartate aminotransferase, which was first introduced in 2011.

* Not all patients were tested for all analyses.

**Figure 2:**
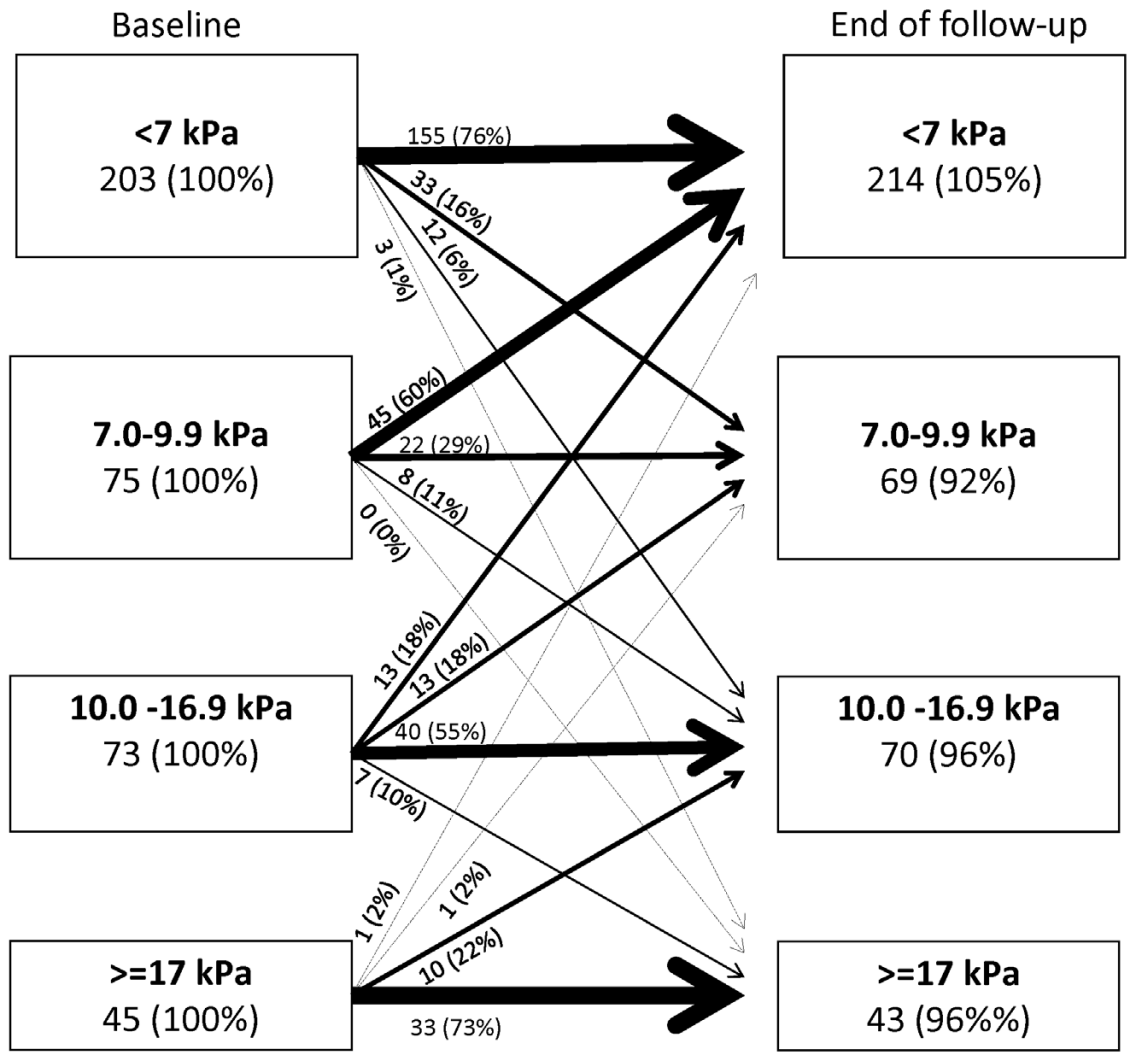
Changes in liver stiffness measurements over time among 396 untreated hepatitis C patients. Percentage values indicate percent of baseline group.

**Figure 3:**
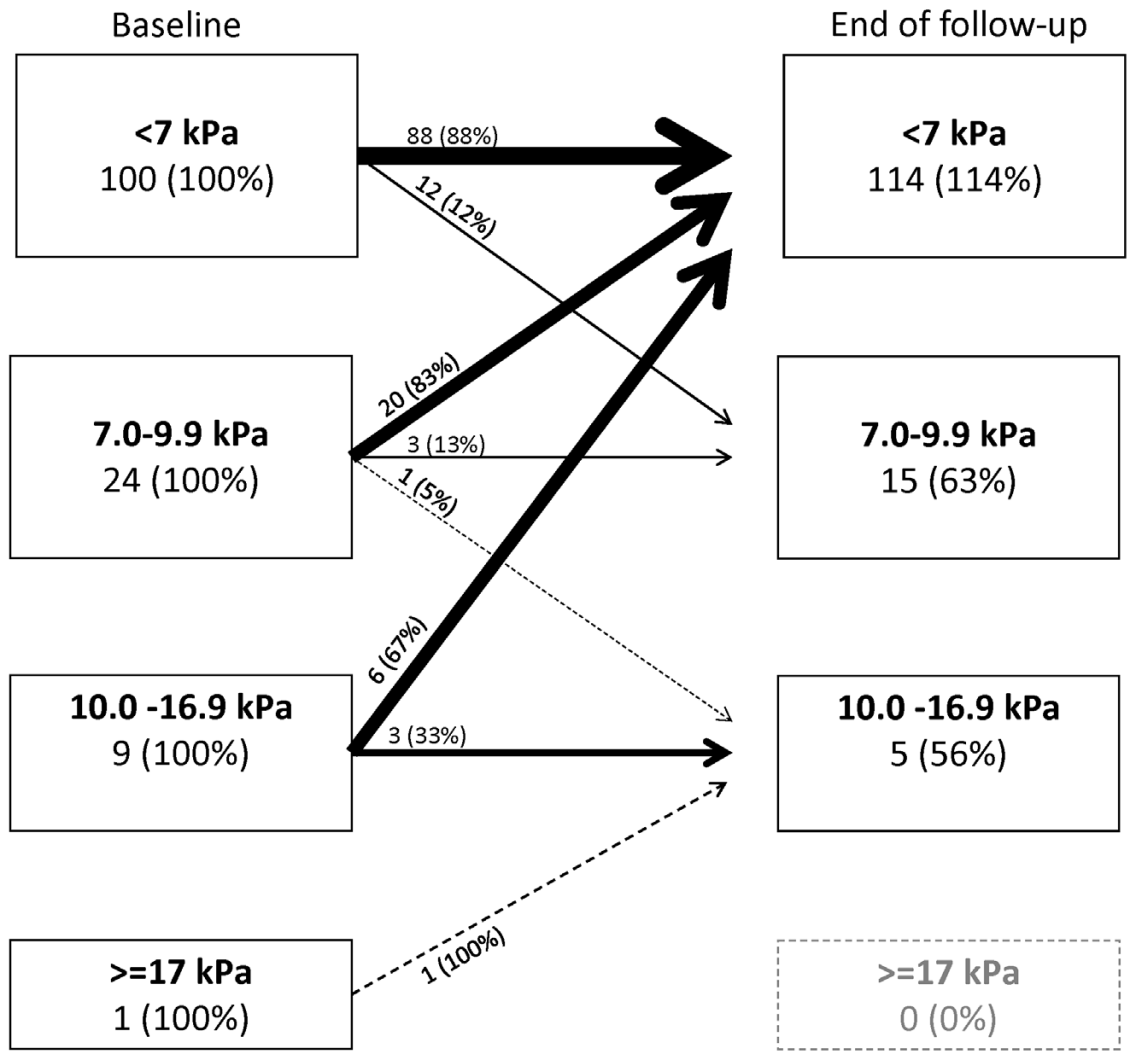
Changes in liver stiffness measurements over time among 134 untreated hepatitis B patients. Percentage values indicate percent of baseline group.

At first examination, the distribution of the predefined LSM categories was 57.8% <7 kPa, 16.1% 7.0–9.9 kPa, 14.1% 10–16.9 kPa, and 12.1% ≥17 kPa. The median number of LSMs performed was 3 (IQR, 2–4; range, 1–17). In the LSM natural history cohort, 315 patients were excluded (Figure 1). Among the remaining 530 patients, 93.6% (496) were treatment naïve and 6.4% (34) had been treated without success more than one year prior to inclusion. In this cohort, we performed 1926 valid scans during a median follow-up of 36 months, corresponding to a total of 1519 person years (PY) of observation (Table 1). Median LSM values were 6.6 kPa at the first examination, and 6.4 kPa at the last examination (p<0.001). Although we observed no clinically relevant change in the overall median LSM over time, a high proportion of individual patients changed LSM category during follow-up, including 30% (40/134) of hepatitis B patients and 36.9% (146/396) of hepatitis C patients (Table 3, Figures 2 and 3). Change in LSM category was most common for patients with an initial LSM value of between 7 and 10 kPa, among whom 83% (20/24) of HBV patients and 60% (45/75) of HCV patients had values below 7 kPa at the end of untreated follow-up (Figures 2 and 3).

**Table 3:** Change between first and last liver stiffness measurements among 530 untreated patients who were monoinfected with viral hepatitis B or C and followed for >3 months.

	No change	Increase	Decrease	Total
First LSM (kPa)	n (%)	n (%)	n (%)	n (%)
**Hepatitis C**				
2.0–6.9	170 (83.7)	33 (16.3)	-	203 (100)
7.0–9.9	41 (54.7)	7 (9.3)	27 (36.0)	75 (100)
10.0–16.9	29 (39.7)	15 (20.5)	29 (39.7)	73 (100)
17.0–75.0	13 (28.9)	10 (29.4)	22 (48.9)	45 (100)
Total	253 (63.9)	15.9)	78 (19.7)	396 (100)
**Hepatitis B**				
2.0–6.9	93 (93.0)	7 (7.0)	-	100 (100)
7.0–9.9	6 (25.0)	1 (4.2)	17 (70.8)	24 (100)
10.0–16.9	1 (11.1)	2 (22.2)	6 (66.7)	9 (100)
17.0–75.0	1 (100.0)	0 (0.0)	0 (0.0)	1 (100)
Total	101 (74.4)	10 (13.3)	23 (12.2)	134 (100)

Change defined as: a difference of >20% of the primary measurement and of >2 kPa, with one measurement >7 kPa.

Among patients with normal initial LSM values (≤7 kPa), 76% of HCV patients and 88% of HBV patients also had normal values at the end of follow-up. However, among the HCV patients whose first and last examination values were both in the normal range, 16% (25/155) had one or more LSM value of >7 kPa during the follow-up period, and 3% (4/155) had one or more LSM value of >10 kPa (none presented an LSM value of >17 kPa). Among the HBV patients with normal first and last LSM examination results, 9% (8/88) had one or more LSM values of >7 kPa, but none were above 10 kPa. Among untreated HCV patients, 10% (41/396) showed a persistent and significant increase in LSM values, corresponding to an incidence rate of 3.4/100 PY (95% CI, 2.5–4.7). Among untreated HBV patients, 4% (6/134) showed a persistent and significant increase in LSM, corresponding to an incidence rate of 1.5 (95% CI, 0.6–3.3)/100 PY.

During the study period, 105 biopsies were performed among the 845 patients, corresponding to 12% of the population (16% of HCV patients and 8% of HBV patients). These biopsies revealed significant fibrosis (≥F2) in 53%, and cirrhosis in 23% of biopsied patients. Among the 54 untreated patients subjected to biopsy, a cut-off of 17 kPa correctly classified 96% as having cirrhosis (sensitivity, 0.92; specificity, 0.95; likelyhood ratio of a positive test (LR+), 19; likelyhood ratio of a negative test (LR−), 0.1).

### Cirrhosis

Among the 845 patients, 132 (16%) were classified as having cirrhosis—of whom 51 (39%) were diagnosed prior to the first LSM, 56 (42%) within 6 months of the first LSM, and 25 (19%) during follow-up. Prior to the introduction of LSM, 67% of cirrhosis diagnoses were made based on biopsy and 33% based on clinical signs of decompensation. After the introduction of LSM, 61 patients (75%) were diagnosed with cirrhosis based on an LSM value of ≥17 kPa, 5 (6%) based on clinical signs, and 15 (19%) by biopsy. In all cases where cirrhosis was diagnosed by clinical signs alone, this diagnosis occurred during the initial workup (the 6-month lead-in phase). During the subsequent follow-up, no patient was diagnosed with cirrhosis based on clinical signs. Excluding the 6-month lead-in phase after the first LSM, the incidence of cirrhosis was 1.0/100 PY (95% CI, 0.6–1.4).

### Decompensated liver disease

Overall, 103 liver-related complications occurred in 67 of 132 patients with cirrhosis (51%). Of these complications, 58 (56%) were observed among 32 patients (24%) during follow-up, corresponding to an 11.5/100 PY incidence of complications among patients with cirrhosis. During follow-up, liver-related complications occurred only among patients with a previous diagnosis of cirrhosis and who were infected with HCV (including one patient co-infected with HBV and HIV). Patients diagnosed with cirrhosis based on an LSM value of ≥17 kPa without biopsy had the same risk of complications during follow-up (incidence of 11.1/100 PY) as patients with biopsy-proven cirrhosis (12.1/100 PY; incidence rate ratio (IRR), 0.92; 95% CI, 0.4–2.0; p = 0.8).

### Mortality

Among the 845 patients, 56 deaths occurred, corresponding to 1.9/100 PY of observation (95% CI, 1.4–2.4). Fifty of these deaths occurred among the 586 hepatitis C monoinfected patients, one of whom received a liver transplant, corresponding to 2.4/100 PY (95% CI, 1.7–3.1). The liver-related mortality was 0.65/100 PY (0.35–1.08) (13 deaths and one liver transplant) and death by drug-related causes was 0.51/100 PY (0.25–0.91) (11 deaths) (Table 3). Of the 13 liver-related events, 11 occurred among HCV patients with an initial LSM value of ≥17 kPa. The remaining two patients registered as death from liver-related causes had initial LSM values of <7 kPa. However, one was probably a registration error, as the repeat LSM value was 65 kPa and the patient had biopsy-proven cirrhosis prior to the first examination. The other patient had F1 fibrosis in a liver biopsy performed in 2004, 3 LSM values of <5.5 kPa during 2007–2010, and no clinical or biochemical signs of liver disease at last follow-up. The patient died 11 months later with no history of preceding illness. No autopsy was performed, and the death certificate was filled out by a private practitioner who stated liver coma to be the immediate cause of death, and cirrhosis due to hepatitis C as the underlying cause of death. We consider this cause of death unlikely. Excluding this patient, the liver-related mortality was 0.60/100 PY (12 deaths and one transplant) overall, and 2.5/100 PY for patients with cirrhosis.

Among the 217 monoinfected hepatitis B patients, 5 died, corresponding to 0.73/100 PY (0.23–1.69). No liver-related deaths were observed (97.5% CI, 0–0.43). Nine patients (4%) had an initial LSM value of >17 kPa, compatible with cirrhosis. Of these 9 patients, 7 started treatment, of whom 4 had LSM values of <17 kPa at end of follow-up (two with values in the normal range).

We found a high predictive value of initial LSM for liver-related deaths among the 845 patients in our cohort. The overall area under the receiver operating characteristics curve (AUROC) was 0.86, and the AUROC calculated excluding the two patients that we believe were not correctly classified was 0.96. No liver-related deaths occurred in patients with an initial LSM value of <17 kPa. In contrast, the prediction of overall mortality based on LSM was low (AUROC of 0.64) reflecting that only 23% (13/56) of the deaths observed in our cohort were considered to be liver related.

## Discussion

The present single-center study evaluated the use of LSM to monitor liver fibrosis in a routine clinical setting over a five-year period with complete mortality follow-up. We found a significant spontaneous decrease in LSM values during untreated follow-up among patients with an initial LSM of <10 kPa. In contrast, patients with LSM values of ≥17 kPa had a clinically significant incidence of liver-related complications regardless of whether cirrhosis had also been diagnosed by biopsy.

Using our present definition of significant and persistent fibrosis progression (20% increase of a minimum of 2 kPa, with one value above 7 kPa, and all elevated values documented at least twice), such progression was observed in 3% of the untreated hepatitis C patients and 2% of the hepatitis B patients per year. Previous studies have reported similar rates of progression in HCV-infected drug users (median 3% progression during 1.8 years of follow-up) and among hemophiliacs (4.1/100 PY), although these studies did not use the same definition of fibrosis progression as our study [20], [21]. The incidence of cirrhosis in our study was 1/100 PY during follow-up, which is in accordance with results from prior cohort studies of hepatitis C patients [22].

A striking finding of our present study was that many patients showed a decline in LSM values, or had fluctuating values during follow-up (Table 3). This confirms the results from our previous screening study showing that 30% (6/20) of drug users with an initial LSM value of >12 kPa had regressed to a median value of 8.4 kPa after three months. These data are also in agreement with the results reported by Mehta et al., demonstrating that 48% of patients with initial LSM values of between 8–12 kPa had one or more measurement of <8 kPa during follow-up [20], [23]. A study of hemophiliacs showed that 32% exhibited more than 2 kPa of regression during 3.7 years of follow-up [21]. Among hepatitis B patients, Castera et al. [24] reported that 81% (9/11) with an initial LSM value of >7.2 kPa regressed to normal values during 22 months of follow-up, and Kim et al. [25] reported that 30% (34/114) of patients with F3+ in biopsy regressed more than 30% during 13 months. For comparison, studies using repeated biopsy have reported that up to 24% exhibited decreasing fibrosis during 3–4 years of untreated follow-up [24]–[26]. Our present study did not address the causes of the observed variations; however, our findings suggest that clinical decisions, such as treatment initiation, or prediction of prognosis for long-term liver-related complications should not be based on a single LSM measurement.

One major change that occurred at our clinic after LSM introduction was that we no longer observed patients who developed liver decompensation without a previous diagnosis of cirrhosis. Prior to LSM, one-third of our patients with cirrhosis were diagnosed based on decompensation [27]. Among these patients, cirrhosis had not been suspected or the patients had refused to undergo liver biopsy. Our present rates of complications (12.7/100 PY) and liver-related deaths (0.6/100 PY) were comparable to those previously reported in both LSM-based and biopsy-based studies, in which decompensation has been reported as 5–12/100 PY and liver-related mortality as 1/100 PY [18], [28]–[31]. We observed few liver-related complications among hepatitis B patients; however, due to the low number of events, the incidence was not statistically different from that among hepatitis C patients. This is in contrast to Asian studies that have reported a similar or higher prevalence of cirrhosis and liver-related events among HBV patients compared to HCV patients [32]–[34]. Compared to the HCV patients in our study, the HBV patients showed fewer risk factors associated with cirrhosis; only 9 HBV patients had cirrhosis and 20% of all HBV patients received continuous nucleoside treatment during follow-up.

All of the above cited studies, as well as studies in hepatitis B patients, are in agreement with our finding that LSM was a strong predictor for liver-related events, with optimal cut-offs of 13–20 kPa for predicting events, and very low risk associated with values below 10 kPa [35]. This association was confirmed in a recent meta-analysis of LSM as a predictor of complications, showing a 22% increase in mortality per kPa increase in baseline LSM [36]. However, they did not report a lower threshold for complications.

Our study has several weaknesses. We focused on LSM examinations and did not include clinical or laboratory tests in the evaluation. These parameters would have improved patient characterization and probably would have increased the precision of our estimates, for instance hepatitis C patients had significantly higher liver enzymes than hepatitis B (Table 2). Several studies have found ALT to be an independent predictor of LSM value regression, and it has been suggested that LSM be combined with serological markers of fibrosis in order to increase diagnostic accuracy [3], [37]–[39]. However the addition of laboratory data would have also limited the interpretation of LSM performance alone. The regression towards normal among patients with initial LSM values in the 7–10 kPa range could have partly been due to the fact that repeat scans were performed in the fasting state. However, after detection of an elevated LSM value, the patient was followed in the fasting state, as has been our standard practice since 2008. Additionally, the XL probe has been shown to produce values up to 1 kPa lower when compared to the median probe [40]. However, we used the XL probe in cases where the M probe did not give valid results, and thus very few patients had an initial valid scan with the M probe and a scan with the XL probe at the end of follow-up.

## Conclusions

Over the past years, LSM has been widely introduced as a screening tool for fibrosis, with patients showing an LSM value above the normal range potentially being considered candidates for antiviral treatment. Our present study showed that the LSM values of many of these patients returned to within the normal range during untreated follow-up. Furthermore, no cases of liver-related death or morbidity were observed in patients with moderately and intermittently elevated LSM values. Our findings suggested that clinical decisions should not be based on a single elevated LSM measurement. Furthermore, our present results indicated that patients with LSM values of ≥17 kPa should enter surveillance for complications of cirrhosis.
